# Deep learning to quantify care manipulation activities in neonatal intensive care units

**DOI:** 10.1038/s41746-024-01164-y

**Published:** 2024-06-27

**Authors:** Abrar Majeedi, Ryan M. McAdams, Ravneet Kaur, Shubham Gupta, Harpreet Singh, Yin Li

**Affiliations:** 1 Department of Biostatistics and Medical Informatics, School of Medicine and Public Health, University of Wisconsin-Madison, Madison, WI USA; 2 Department of Pediatrics, School of Medicine and Public Health, University of Wisconsin-Madison, Madison, WI USA; 3 Child Health Imprints (CHIL) USA Inc, Madison, WI USA; 4 Department of Computer Sciences, School of Computer, Data and Information Sciences, College of Letters and Science, University of Wisconsin-Madison, Madison, WI USA

**Keywords:** Paediatrics, Computer science

## Abstract

Early-life exposure to stress results in significantly increased risk of neurodevelopmental impairments with potential long-term effects into childhood and even adulthood. As a crucial step towards monitoring neonatal stress in neonatal intensive care units (NICUs), our study aims to quantify the duration, frequency, and physiological responses of care manipulation activities, based on bedside videos and physiological signals. Leveraging 289 h of video recordings and physiological data within 330 sessions collected from 27 neonates in 2 NICUs, we develop and evaluate a deep learning method to detect manipulation activities from the video, to estimate their duration and frequency, and to further integrate physiological signals for assessing their responses. With a 13.8% relative error tolerance for activity duration and frequency, our results were statistically equivalent to human annotations. Further, our method proved effective for estimating short-term physiological responses, for detecting activities with marked physiological deviations, and for quantifying the neonatal infant stressor scale scores.

## Introduction

Worldwide, preterm birth impacts approximately 1 of 10 newborns, accounting for nearly 15 million neonates annually^1^. Complications from preterm birth have emerged as the leading cause of death among children below the age of 5^2^. A substantial proportion of these premature newborns, estimated at 40–60%^3,4^, require admission to neonatal intensive care units (NICUs) specifically designed for critically ill infants. Throughout their NICU stay, which may span several weeks^5^, these newborns undergo numerous distressing interventions and painful procedures, experiencing an average of 23 daily acute stressful events and 43 h of stressful exposure during their first 4 weeks of NICU hospitalization^6^. These early-life stressors are shown to be deleterious to the neonatal nervous system that is immature and rapidly developing^7^. Increasing evidence suggests that significant exposure to pain and stress can lead to a significantly increased risk of neurodevelopmental impairments^8,9^, which may have long-term effects extending into childhood and even adulthood^6,10,11^.

Quantitative assessment of cumulative neonatal stress in the NICU allows for the monitoring of the early-life stressors and facilitates the mitigation of their consequences. Existing approaches for neonatal stress assessment in NICUs are inadequate in clinical environments. The commonly adopted Neonatal Infant Stressor Scale (NISS)^12,13^ relies on manual counting of procedures and interventions, in order to measure a neonate’s cumulative exposure to both acute and chronic stressors. However, implementing the NISS in clinical settings presents major challenges. Health professionals must simultaneously care for many neonates and complete various tasks while monitoring and documenting the procedures and interventions. Further, NISS scores, derived from a perspective survey, are assigned based on the occurrence of procedures and interventions and the age of the neonates. The resultant measurements overlook the nuances of care quality, thereby presenting a rather simplistic assessment that is subject to considerable variability^14^. There is thus an unmet need to develop a more robust and practical method for quantifying neonatal stress in NICUs.

Readily available sensor data, such as physiological signals and bedside video footage, in conjunction with machine learning techniques, offer a promising solution for automated, continuous, objective, and efficient quantification of neonatal stress in NICUs. Recent research has investigated machine learning in NICUs for non-contact monitoring of vital signs using videos^15^, and for detecting stress related to bradycardia (low heart rate) using physiological recordings^16^. While these physiological effects, such as changes in heart rate and breathing patterns, are often linked to neonatal stress, they can also be influenced by routine care activities (e.g., diaper changes) or common NICU conditions (e.g., apnea). Consequently, relying solely on these indicators is insufficient for accurately quantifying stress levels. Video-based, automated monitoring of patient mobilization activities has been previously studied in adult ICUs^17,18^. A prior work^19^ explored automated classification of manipulation activities in pre-selected video clips, and associated the results with physiological parameters. However, their study necessitated manual annotation of activity onsets and offsets in the video at deployment time, and did not evaluate the quantification of these activities (e.g., the duration or frequency), rendering it both labor-intensive and impractical for clinical settings.

A key research focus lies in the quantification of stress-inducing procedures and interventions in NICUs, with the aim of accurately measuring and appropriately mitigating the resulting cumulative stress. This study specifically targets the quantification of certain neonatal care manipulation activities, such as diaper changing, tube feeding, and patting, which are frequently performed in NICUs^20^ and recognized as moderate stressors^12^. However, these activities are often inadequately documented in clinical practice and remain unaddressed in the existing literature. To fill this gap, the goal of this study is to develop and evaluate a deep learning method that analyzes bedside video and integrates physiological data, in order to assess the frequency, duration, and short-term impact of care manipulation activities in NICUs.

## Results

Based on a total of 289 h of video recordings and physiological data within 330 sessions collected from 27 neonates receiving care in the NICU, our deep learning method was trained to detect care manipulation activities present in the videos, to quantify their occurrence over time, and to further integrate physiological signals for assessing their short-term impact. Our method was evaluated with five-fold cross validation for quantifying the duration and frequency of representative care manipulation activities (diaper change, tube feeding, and patting) and for assessing their short-term physiological responses. Our evaluation demonstrated clinically acceptable performance. For the quantification of duration and frequency, with a tolerance of 13.8% relative error, the method achieved results that were statistically equivalent to human annotations. For the assessment of impact, the method showed a high level of consistency for estimating immediate and short-term physiological responses in comparison to human annotations and attained an average precision of 89.5% and an average recall of 72.7% for detecting activities with marked physiological deviation. Further, our method demonstrates promising results for automated quantification of NISS scores^12^. With a tolerance of 12.7% relative error, the NISS scores for diaper change predicted by our method were comparable to those from human observations.

### Performance for quantifying the duration and frequency of care manipulation activities

**Fig. 1:**
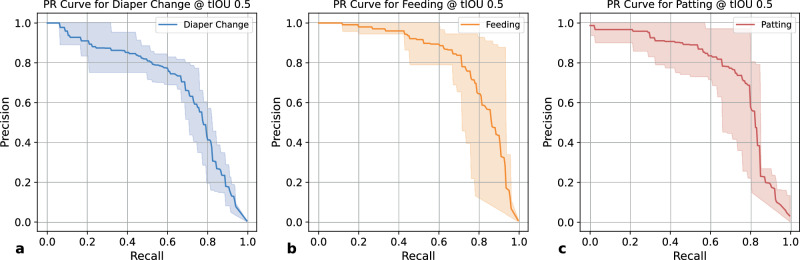
Activity detection results. Precision-Recall (PR) curves for detecting three manipulation activities (diaper change (**a**), feeding (**b**), and patting (**c**)) at a tIOU threshold of 0.5. Lighter bands indicate the variations across all five test splits.

For localizing and recognizing care manipulation activities in the video, our method achieved an average precision of 81.7% with Standard Deviation (SD) = 4.5% and an average recall of 73.9% (SD = 7.1%) across three manipulation activities (diaper change, feeding, and patting), when considering a temporal Intersection over Union (tIoU) threshold of 0.5. tIoU measures the temporal overlap between a predicted event and a human annotated event. A threshold of 0.5 requires a predicted event having a major overlap with a human annotated event in order to be considered as a true positive. The precision-recall curves are shown in Fig. 1. Detailed results including additional metrics of F1 scores and average precision (AP)^21^ are presented in Supplementary Table 1. An additional experiment was carried out to evaluate the generalizability of our method, in which our method was trained on videos from one site (an urban NICU) and tested on videos from a different site (a rural NICU). The results are described in Supplementary Fig. 1.

**Fig. 2:**
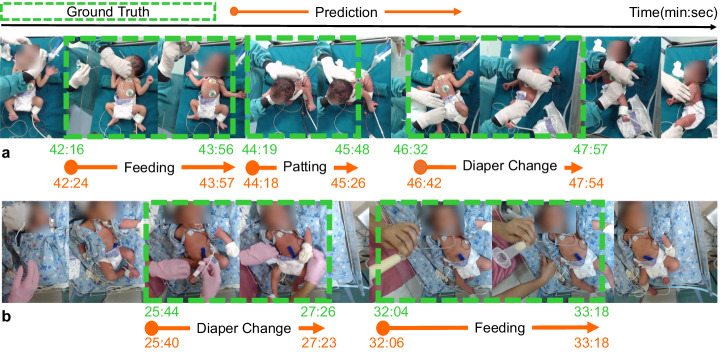
Visualizations of the activity localization and recognition results. For two test videos (**a**, **b**), the starting and ending times (in minute:second) of predicted activities, as well as their predicted labels, are compared against those from human annotations. Faces are blurred to protect privacy.

Visualizations of the activity detection results, in comparison to human annotated ground-truth, are presented in Fig. 2. Diagnostic analyses of the results using the tool from Alwassel et al.^22^ are shown in Supplementary Figs. 2 and 3. The analyses showed low false negative rates and high average precision across the board. A performance degradation was observed for extreme short (<30 s) and long activities (>360 s), with increased false negative rates and decreased mean precision. The analyses also suggested that inaccurate temporal boundaries of the predicted activities were the major source of errors for false positives. Additional results of using a tighter tIoU threshold (0.75) are included in Supplementary Fig. 4 and Supplementary Table 2.

**Fig. 3:**
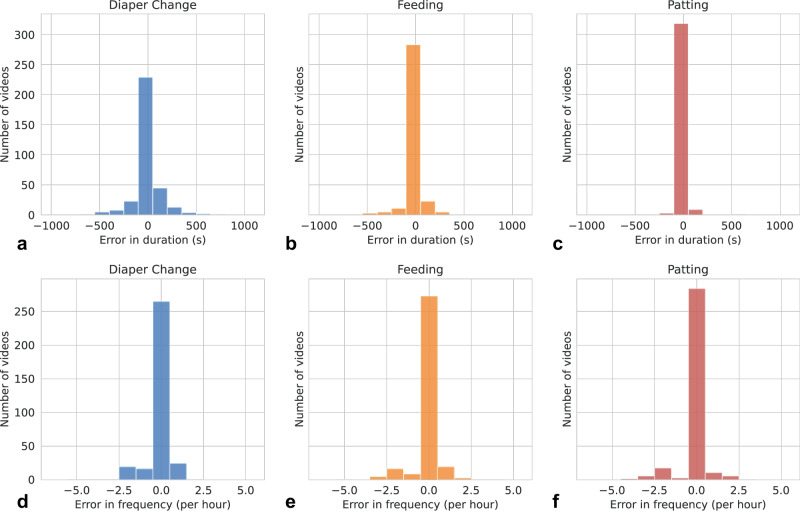
Prediction error plots. Histograms of prediction errors in duration and frequency for diaper change (**a**, **d**), feeding (**b**, **e**) and patting (**c**, **f**) activities. Top: Errors in duration. Bottom: Errors in frequency. Errors are reported across all five splits.

Video-based activity detection results were further accumulated to quantify the duration and frequency of care manipulation activities. A paired two one-tailed t-test was performed to compare the algorithmic predicted activities and the human annotated ones. At 95% confidence interval (*p*-value < 0.05), the relative error in duration with respect to the average duration was 9.2%, 13.8% and 10.4% for diaper change, feeding, and patting, respectively. Similarly, the relative error in frequency was 13.2%, 10.6% and 5.9% for diaper change, feeding, and patting, respectively. The results suggested that with a tolerance of 13.8% relative error, the predicted duration and frequency were *statistically equivalent* to those from human annotations. Histograms of prediction errors in duration and frequency across three activity categories are shown in Fig. 3.

### Performance for assessing the short-term impact of care manipulation activities

**Fig. 4:**
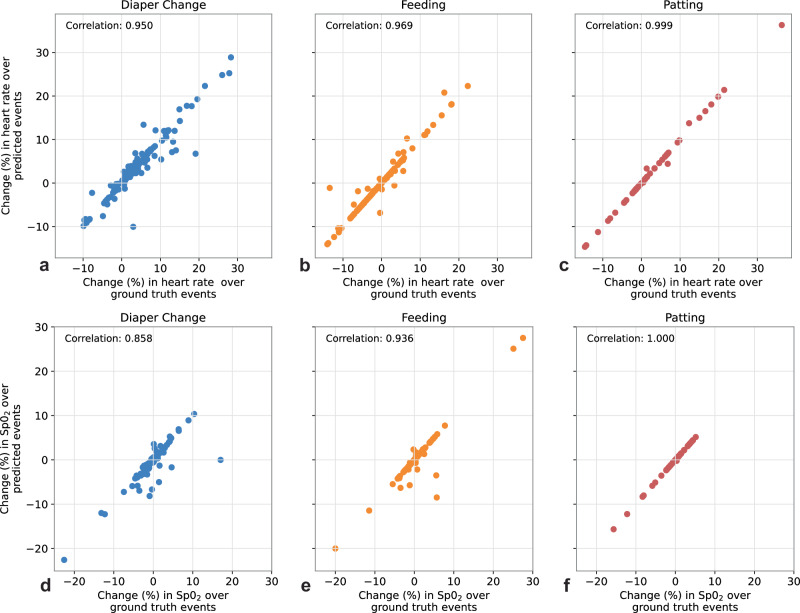
Quantifying changes in heart rate and SpO_2_ during manipulation activities. Scatter graphs that compare relative changes (%) in heart rates (top) and SpO_2_ levels (bottom) between care manipulation activities automatically detected from videos (vertical axes) and their corresponding human annotated activities (horizontal axes). Plots are shown across diaper change (**a**, **d**), feeding (**b**, **e**), and patting (**c**, **f**) activities over five splits.

Physiological signals, including heart rates and oxygen saturation levels (SpO_2_), were combined with video-based activity detection results to evaluate the physiological responses to care manipulation activities. Relative changes in heart rates and SpO_2_ levels noted during automatically video-detected activities were compared to those noted during manually annotated, true positive activities. The relative changes, as a proxy of immediate physiological responses, were calculated as the ratio of change in the average heart rates or SpO_2_ levels before the activity (baseline) and during the activity. For heart rates, the Pearson correlation coefficient was 0.950, 0.969, and 0.999 for diaper change, feeding, and patting, respectively. For SpO_2_ levels, correlation coefficient was 0.858, 0.936, and 1.00 for diaper change, feeding and patting, respectively. The results suggested a strong correlation between immediate physiological responses derived from automatically detected activities and from their corresponding human annotated activities. A comparison of relative changes in physiological signals from detected activities and from human annotated activities are presented in Fig. 4.

**Fig. 5:**
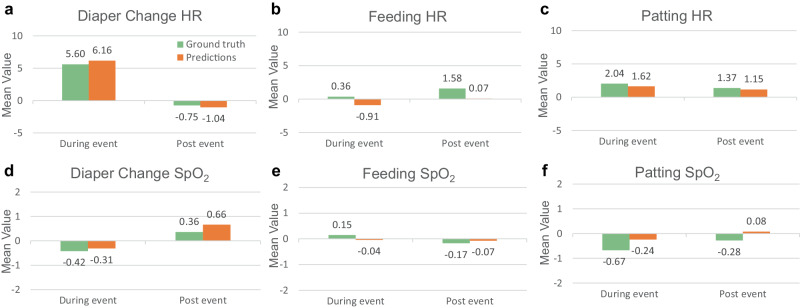
Changes in heart rate and SpO_2_ before, during and after manipulation activities. Differences in average heart rates (**a**–**c**) and SpO_2_ levels (**d**–**f**) prior to (before) and during an activity, or prior to (before) and subsequent to (post) an activity, when evaluated using either algorithm predicted activities or human annotated activities. Results are reported across three manipulation activities and averaged over five splits.

To further evaluate short-term physiological responses of care manipulation activities, paired t-tests were employed to examine the differences in physiological signals prior to (10 min before), during, and subsequent to (10 min post) an activity. Average heart rate and SpO_2_ level prior to an activity were considered as baselines and compared against those during, and subsequent to an activity. Statistically significant differences were observed when comparing heart rates prior to and during a diaper change activity using either algorithm predicted activities or human annotated activities. Specifically, the average difference (beats per minute) in heart rates prior to and during a diaper change activity was 6.16 (*p*-value < 0.01) with algorithmic predictions and 5.60 (*p*-value < 0.01) with human annotated activities. No major differences were identified for other activities (feeding and patting) or settings (before vs. during and before vs. post for SpO_2_). Detailed results are summarized in Supplementary Table 3. Figure 5 illustrates the variation in physiological responses across different manipulation activities, as assessed by both algorithm predictions and human annotations. This notable congruence between algorithmic predictions and human annotation suggested that algorithmic predictions can effectively capture the group-level average statistics of short-term physiological responses with a degree of accuracy comparable to human annotations. Further, these results confirmed the clinical observation that diaper change often involves more intense manipulation than those gentler and shorter ones in feeding and patting, leading to increased heart rate in short term, as previously reported^19^.

Our deep learning method’s ability to automate the detection of care manipulation activities can be demonstrated through its analysis of video and corresponding physiological signals. This is particularly notable in situations with marked deviation in physiological responses. For example, certain approaches to diaper changing in premature neonates can trigger a stress response, marked by elevated heart rates or reduced SpO_2_ levels^23^. Our method was further evaluated on the task of detecting activities with marked physiological deviation. An activity was classified as inducing a significant physiological deviation if it elicited a relative change of at least 15% in heart rate or SpO_2_ levels, compared to baseline measurements obtained prior to the commencement of the activity. Following a similar evaluation protocol used in the “Results on activity detection” section, and applying a tIOU threshold of 0.5, our method achieved an average precision of 89.5% (SD = 17.4%) and an average recall of 72.8% (SD = 25.5%) across three different care manipulation activities. A corresponding precision-recall curve is illustrated in Supplementary Fig. 5. The observed variability in the results can be attributed to the infrequent occurrence of these activities (only 36 instances across all test splits) and their uneven distribution across the test splits.

Our method was further evaluated for the automated quantification of the NISS scores^12^. Our evaluation specifically focuses on NISS scores for diaper change. This partial NISS score measures a neonate’s cumulative exposure to stress associated with diaper change, and assigns a score based on the frequency of diaper change and the age of a neonate. Scores derived from algorithm-predicted diaper change frequency were compared to those from human-annotated frequency for all videos. A paired two-one sided t-test was performed. The results showed that with a tolerance of 11.8% relative error, the algorithm-predicted scores were comparable to the human-annotated scores (*p*-value < 0.05).

## Discussion

This paper presented a deep learning method to quantify care-related activities (diaper change, feeding, and patting) in the NICU. Leveraging multi-modal sensor data and deep learning, our method accurately detected activities, measured their duration and frequency, and assessed neonatal physiological responses. With a tolerance of 15% relative error, the method demonstrated comparable accuracy to human annotations for the measurement of activity duration and frequency. The deep learning method also showed highly consistent results for estimating immediate and short-term physiological responses in comparison to human annotations. Further, the method demonstrated encouraging capability in detecting activities with marked physiological deviations, and in quantifying the NISS scores.

Other factors beyond stress may explain the marked physiological deviation. For example, increased physical activity during patting could result in elevated heart rate, rather than stress itself. Environmental stressors like bright lights or loud noises can also prompt physiological responses such as changes in heart rate and oxygen saturation compared to baseline^24^. To better determine the underlying cause of these responses, future work could explore video-based neonate pose estimation to assess posture and movement^25,26^. Additionally, incorporating extra sensing modalities like photometers to measure light levels and sound sensors for noise could help account for environmental contributors. With enhanced sensing of both neonatal activity and ambient conditions, the actual factors driving physiological changes can be more reliably identified.

Automating the quantification of care-related activities is an essential step toward continuous, objective neonatal stress assessment in the NICU. Manual documentation of activities is currently required, a challenging task given frequent staffing shortages, distractions, fatigue, and the inability to constantly observe each neonate. Moreover, existing approaches like the NISS neglect care quality considerations. The current study demonstrated new capabilities to quantify activity duration, frequency, and associated vital sign changes associated with different care activities in the NICU.

Further, our study suggests the feasibility of an automated Neonatal Infant Stress Score (NISS)^12^ designed to complement traditional pain observations in assessing infant stress during NICU stays. With further advancements, a video-based machine learning approach could facilitate real-time quantitative NISS assessments alongside qualitative pain observations by nurses. This synergy could enhance clinicians’ ability to identify various infant stressors, especially those masked by analgesia or sedation. Given the existing variability in stress assessments among NICU clinicians, an automated, machine learning approach to assign stress scores, like the NISS, promises a standardized method to promote uniform care practices and facilitate systematic research into the effects of stress on infant development and health outcomes.

This study has limitations including the small activity set and dataset from just two hospitals, which may restrict the method’s performance and generalizability. More extensive datasets could improve robustness. Additionally, the method struggled with very brief (<30 s) and long (>360 s) activities, which could be problematic for complex NICU routines. Upon inspection, short activities often excluded the full image of the neonate, while long activities contained disruptions. Recent advances in computer vision and machine learning offer potential solutions. For instance, learning from large-scale unlabeled video^27,28^ could capture diverse visual patterns. Incorporating audio information^29,30^ could help with the recognition when visual information alone is insufficient.

**Fig. 6:**
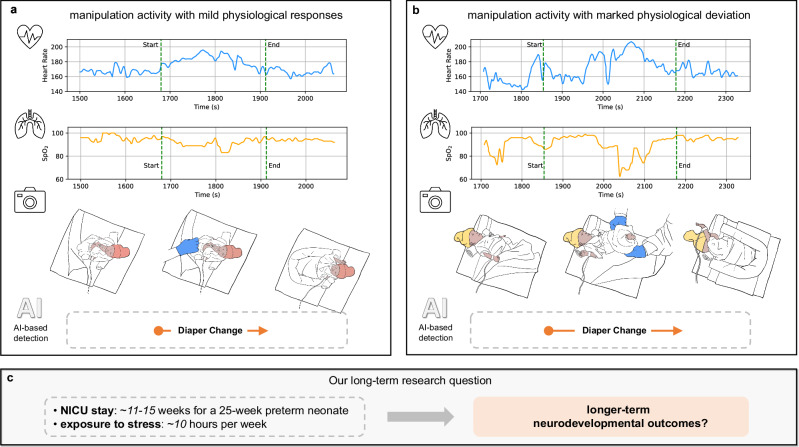
NICU stressors and multi-modal sensing. A schematic illustration of exemplar NICU stressors and their responses captured by multi-modal sensing (**a**, **b**), with a highlight of our long-term research question (**c**).

Interest has soared in developing AI strategies for the NICU^31^. AI aggregation and analysis of multi-modal data can provide clinically relevant information to steer decision-making, advance evidence-based practices, bolster patient safety, and predict short and long-term outcomes. This study offers an automated system to standardize documentation of neonatal stressors and identify distressing activities, providing objective monitoring of factors contributing to neonatal stress. Our approach can free up medical staff time, enhance decision-making, and ultimately improve neonatal health outcomes. Real-time feedback from automated analyses could enable around-the-clock neonatal stress monitoring, paving the way for swift intervention adjustments. This approach, as illustrated in Fig. 6, in turn, lays the groundwork for unraveling the longer-term neurodevelopmental outcomes associated with neonatal stress. Future work should focus on gathering more diverse data across multiple centers and assessing the method’s clinical integration through larger multicenter controlled trials to validate its performance and utility in various clinical settings.

## Methods

### Study design and participants

To develop and evaluate our method, a multi-modal dataset of neonates undergoing routine care procedures in NICUs was collected and considered. The dataset, comprised of video recordings and their associated physiological data, subsumed data points used in Singh et al.^19^ and included a substantial extension (an additional 218 h of recordings).

**Fig. 7:**
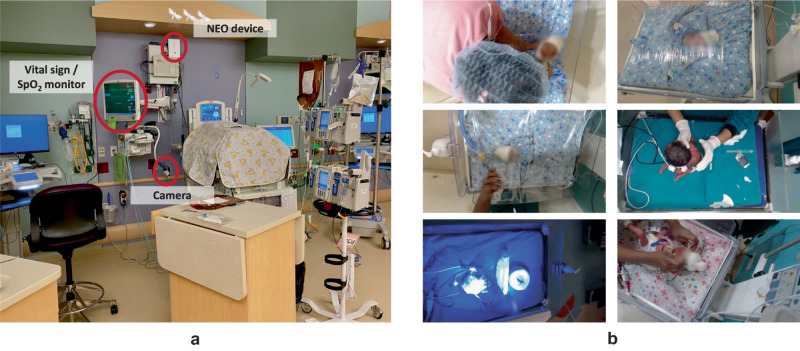
Illustration of our setup and sample video frames. **a** Data recording setup in a NICU. **b** Sample video frames with faces blurred for privacy.

The NEO device with a camera module^32^ was used to collect the video and physiological data. A NEO device was installed on each bed and recorded videos from the camera module, as well as heart rates and SpO_2_ levels from existing monitoring devices in the NICU. The camera module consisted of a Logitech C920 mounted on a flexible tube, allowing for the adjustment of camera position. The study was designed to minimally interfere with the clinical workflow. The only instruction provided to the nursing staff and other attendants was to ensure that the camera was oriented towards the neonate during their routine care procedures. The resulting videos captured activities from various lighting conditions (e.g., different time of a day) and viewing angles (e.g., different positioning of the camera with respect to the neonate). The data recording setup and sample video frames are shown in Fig. 7.

All data were collected from a level III 22 bed urban NICU and level II-b 17 bed rural NICU in India. The typical staff in the NICUs consisted of 3 neonatologists with doctorate degrees in neonatal sciences, 3-4 medical residents, and 18-20 nurses. 27 neonates were included in the study with an average age of 33.0 weeks. The Institutional Review Board from the Apollo Cradle & Children hospital (Moti Nagar, Delhi, India) and from the Kalawati Hospital (Rewari, India) approved this study and waived the requirement for informed consent. The electronic health records of the neonates were de-identified in accordance with Health Insurance Portability and Accountability Act regulations, and the research was conducted in compliance with relevant guidelines. For figures included in this paper and its additional information that involve images of neonates with blurred faces, written consents were obtained from the parents of eligible neonates.

### Data statistics and annotation

Video recordings were captured at a resolution of 1280 × 720 pixels and a rate of 30 frames per second. Physiological data were recorded at various rates ranging from 1 reading per minute to 1 reading per second, depending on the monitoring device in the NICU units. A manual inspection was performed to select videos in which the neonate is clearly visible and at least of one manipulation activity is observed, leading to a dataset of 330 videos (average length 52.5 min) with their associated physiological data that last 288.8 h in total. Two trained annotators were further tasked to independently mark the start and end times for every instance of diaper change, feeding, and patting events in the video. A further verification was performed to address the discrepancy in the annotations. A total of 650 care manipulation activities were identified in the video dataset. Due to issues in synchronization and data corruption, 479 out of these 650 activities (corresponding to 19 out of the total 27 neonates) had physiological data synchronized with the video. Details of the dataset are summarized in Table 1.

**Table 1:** Statistics of our dataset consisting of videos and physiological data

Manipulation activity	Sample size	Average duration (seconds)	Average frequency (counts per hour)
Diaper change	230	228.07	1.10
Tube feeding	217	113.03	1.14
Patting	203	32.79	1.17
All	650	128.68	1.13

### Deep learning for video analysis

**Fig. 8:**
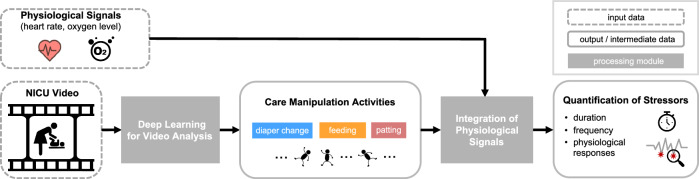
Overview of our method. The video data processed by deep learning is integrated with physiological data to quantify the stressors for a neonate in a NICU.

Central to our approach lies in a deep learning method for temporal activity localization — the ability to recognize the occurrence of care manipulation activities in a video, and localize their corresponding onsets and offsets. Our deep learning approach combines methods for video representation learning and for temporal activity localization. A flow chart of our method is illustrated in Fig. 8.

To represent an input video, a convolutional neural network (SlowFast^33^) pre-trained on a large-scale Internet video dataset (Kinetics^34^) was considered to extract video features. SlowFast is well suited for analyzing NICU videos; it has been widely adopted for video understanding, and demonstrates reliable efficacy across a diverse range of tasks, including the recognition of human activities^33^, the monitoring of animal behaviors^35^, and the analysis of driving scenarios^36^. To bridge the gap between NICU videos and the Internet videos used for pre-training, a transfer learning approach was further employed to adapt the pre-trained model for NICU videos. Specifically, the SlowFast model was fine-tuned using labeled video clips of care manipulation activities sampled from the videos in the training set during cross-validation. Each clip spanned 2.67 s containing 32 frames sampled at 12 Hz, with each frame randomly cropped to a size of 224 × 224 from a resolution of 512 × 288 (width × height). This fine-tuning step was found helpful to improve the performance of activity detection, as evaluated in Supplementary Table 4. The fine-tuned model was then used to extract clip-level video features for temporal activity localization. Video features were extracted from overlapping clips, each spanning 2.67 s (32 frames sampled at 12 Hz at a resolution of 512 × 288) with a temporal interval of 1.33 s.

To detect activities in the video, a latest method ActionFormer previously developed by our group^37^ was adapted. ActionFormer leverages a Transformer-based model^38^, offers an open-sourced tool for temporal activity localization, and has demonstrated state-of-the-art results on major benchmarks^37^. ActionFormer is deemed appropriate for detecting NICU care manipulation activities due to two main reasons. First, it has been widely adopted to analyze various human activities, ranging from sports and activities of daily living^37^ to nursing procedures^39^, attesting to its versatility and effectiveness. Secondly, it incorporates local self-attention mechanism, and thus supports the efficient processing of hour-long videos, which is common in the NICU. To further justify our method, Supplementary Table 4 compares the results of ActionFormer on NICU videos to those from latest methods designed for temporal activity localization. Technically, the clip-level video features were input to ActionFormer, in which every moment of the video was examined and classified as either one of the care manipulation activities or the background. If a moment was recognized as manipulation activity, the temporal boundary of the activity including its onset and offset was further regressed by the model. The output was a set of detected manipulation activities, each with its label, temporal onset and offset, and a confidence score. The model, as shown in Supplementary Table 5, was learned with human annotated activities on the training set using the AdamW optimization method^40^ for 60 epochs (learning rate of 2e−4 and batch size of 8). Additionally, Supplementary Table 6 presents an ablation study of the model design.

### Integration of physiological signals

To quantify physiological responses of care manipulation activities, heart rates and SpO_2_ levels accompanying the videos were further integrated with video-based activity detection results. The videos and physiological signals were synchronized during recording using the NEO device. Videos without synchronized physiological signals and events lacking physiological readings (due to varying temporal resolution of physiological signals and the duration of events) were excluded from the analysis of physiological responses. Based on the temporal boundary of a detected activity or a human annotated activity, the physiological signals were averaged prior to (as the baseline), during, and subsequent to the activity.

### Quantification of NISS scores

The NISS^12^ considers a wide array of common procedures in NICUs. As a first step, our study explores the quantification of cumulative stress associated with diaper change, regarded as a procedure of moderately stress. The corrected gestational age (CGA) of the neonates was collected and assumed known at the point of each video recording. Videos with CGA > 37 weeks were excluded from the analysis, as the NISS scores are not defined for this age group. A total of 258 videos were analyzed, where two sets of NISS scores for diaper change were computed using algorithm predicted frequency and using human annotated frequency, following^12^. These two set of scores were further compared.

### Evaluation protocol and statistical analysis

For a fair evaluation of our method, a 5-fold cross-validation was adapted. A stratified sampling, accounting for the number of samples in each of the activity categories, was performed to split the dataset into five non-overlapping folds, each with approximately equal number of activities. Details of the dataset splits are described in Supplementary Table 7. For each fold, our method was trained on the remaining 4 folds and evaluated on the current fold. The results were further aggregated across all 5 test splits. Different evaluation protocols, encompassing multiple metrics, were considered for the temporal localization of care manipulation activities, and for the quantification of duration, frequency, and physiological responses of these activities.

For activity detection, a tIoU threshold was considered when matching the predicted activities to human annotated ones, following the standard practice^41,42^. The tIoU between a predicted activity and a human annotated activity was computed as the intersection of the two events divided by their union. A match between a predicted activity and a human annotated activity was determined if the two events shared the same label and their tIoU was larger than the threshold. A prediction was considered as true positive (correct prediction) if the predicted activity can be matched to one of the annotated activities and the corresponding annotation has not been designated to another prediction. Otherwise, the prediction was counted as false positive (incorrect prediction). An annotated event was regarded as false negative (missed event) if no prediction can be matched to the activity. Per-category precision-recall curves were subsequently calculated based on true positives, false positives, and false negatives. These precision-recall curves fully characterize the performance of activity detection. A comprehensive set of metrics derived from the precision-recall curves were evaluated. Precision and recall were summarized based on the best operating point with the highest F1 score on each precision-recall curve and aggregated across all splits. Per-category average precision computed as the area under the precision-recall curve was also reported.

For the quantification of duration, frequency, physiological responses, and NISS scores, target variables (e.g., frequency of diaper change activities) were computed for each video using either algorithmic predictions or human annotations. Variants of paired t-tests were further conducted to compare the results from the two groups.

### Reporting summary

Further information on research design is available in the Nature Research Reporting Summary linked to this article.

### Supplementary information

Supplementary Information

Reporting Summary

## Data Availability

The datasets considered in this study are not publicly available due to the sensitive and identifiable nature of the data, parental consent and restrictions of the ethics protocol to protect the privacy of preterm infants involved in the study.

## Code availability

An open-source custom software is released on https://github.com/abrarmajeedi/DL-to-quantify-manipulation-activities-in-NICUs for detecting and quantifying care manipulation activities in NICU videos, and for integrating physiological signals.

## References

[1] Blencowe H (2012). National, regional, and worldwide estimates of preterm birth rates in the year 2010 with time trends since 1990 for selected countries: a systematic analysis and implications. Lancet.

[2] Perin J (2022). Global, regional, and national causes of under-5 mortality in 2000–19: an updated systematic analysis with implications for the sustainable development goals. Lancet Child Adolesc. Health.

[3] Kim Y, Ganduglia-Cazaban C, Chan W, Lee MJ, Goodman DC (2021). Trends in neonatal intensive care unit admissions by race/ethnicity in the united states, 2008–2018. Sci. Rep..

[4] Basnet S, Adhikari S, Jha J, Pandey MR (2022). Neonatal intensive care unit admissions among preterm babies in a tertiary care centre: A descriptive cross-sectional study. JNMA: J. Nepal Med. Assoc..

[5] Manktelow B, Draper ES, Field C, Field D (2010). Estimates of length of neonatal stay for very premature babies in the UK. Arch. Dis. Child. Fetal Neonatal Ed..

[6] Cong X (2017). The impact of cumulative pain/stress on neurobehavioral development of preterm infants in the NICU. Early Hum. Dev..

[7] Williams MD, Lascelles BDX (2020). Early neonatal pain-a review of clinical and experimental implications on painful conditions later in life. Front. Pediatrics.

[8] Brummelte S (2012). Procedural pain and brain development in premature newborns. Ann. Neurol..

[9] Duerden EG (2018). Early procedural pain is associated with regionally-specific alterations in thalamic development in preterm neonates. J. Neurosci..

[10] Grunau, R. E., Holsti, L. & Peters, J. W. Long-term consequences of pain in human neonates. In *Seminars in Fetal and Neonatal Medicine*, 11-4, 268–275 (Elsevier, 2006). 10.1016/j.siny.2006.02.007 16632415

[11] Beggs S, Currie G, Salter MW, Fitzgerald M, Walker SM (2012). Priming of adult pain responses by neonatal pain experience: maintenance by central neuroimmune activity. Brain.

[12] Newnham CA, Inder TE, Milgrom J (2009). Measuring preterm cumulative stressors within the NICU: the neonatal infant stressor scale. Early Hum. Dev..

[13] Pourkaviani S (2020). Clinical validation of the neonatal infant stressor scale with preterm infant salivary cortisol. Pediatr. Res..

[14] Watterberg KL (2016). Prevention and management of procedural pain in the neonate: an update. Pediatrics.

[15] Villarroel M (2019). Non-contact physiological monitoring of preterm infants in the neonatal intensive care unit. NPJ Digital Med..

[16] Lavanga M (2020). A bradycardia-based stress calculator for the neonatal intensive care unit: a multisystem approach. Front. Physiol..

[17] Yeung S (2019). A computer vision system for deep learning-based detection of patient mobilization activities in the ICU. NPJ Digital Med..

[18] Ma AJ (2017). Measuring patient mobility in the ICU using a novel noninvasive sensor. Crit. Care Med..

[19] Singh H (2020). Machine learning-based automatic classification of video recorded neonatal manipulations and associated physiological parameters: A feasibility study. Children.

[20] Levy J (2017). Impact of hands-on care on infant sleep in the neonatal intensive care unit. Pediatr. Pulmonol..

[21] Everingham M, Van Gool L, Williams CKI, Winn J, Zisserman A (2010). The Pascal Visual Object Classes (VOC) Challenge. Int. J. Comput. Vis..

[22] Alwassel, H., Heilbron, F. C., Escorcia, V., & Ghanem, B. Diagnosing error in temporal action detectors. In *Proceedings of the European conference on computer vision (ECCV)*, 256–272 (Springer International Publishing, 2018).

[23] Koolhaas JM (2011). Stress revisited: a critical evaluation of the stress concept. Neurosci. Biobehav. Rev..

[24] Peng NH (2013). Relationships between environmental stressors and stress biobehavioral responses of preterm infants in NICU. Adv. Neonatal Care.

[25] Groos D, Adde L, Støen R, Ramampiaro H, Ihlen EAF (2022). Towards human-level performance on automatic pose estimation of infant spontaneous movements. Comput. Med. Imaging Graph..

[26] Huang, X., Fu, N., Liu, S. & Ostadabbas, S. Invariant representation learning for infant pose estimation with small data. In *2021 16th IEEE International Conference on Automatic Face and Gesture Recognition (FG 2021)*, 1–8 (IEEE, 2021).

[27] Feichtenhofer C (2022). Masked autoencoders as spatiotemporal learners. Adv. Neural Inf. Process. Syst..

[28] Qian, R. et al. Spatiotemporal contrastive video representation learning. In *Proceedings of the IEEE/CVF Conference on Computer Vision and Pattern Recognition*, 6964–6974 (IEEE, 2021).

[29] Morgado P, Li Y, Nvasconcelos N (2020). Learning representations from audio-visual spatial alignment. Adv. Neural Inf. Process. Syst..

[30] Girdhar, R. et al. Imagebind: One embedding space to bind them all. In *Proceedings of the IEEE/CVF Conference on Computer Vision and Pattern Recognition*, 15180–15190 (IEEE, 2023).

[31] McAdams RM (2022). Predicting clinical outcomes using artificial intelligence and machine learning in neonatal intensive care units: a systematic review. J. Perinatol..

[32] Singh H (2019). Neo-Bedside monitoring device for integrated neonatal intensive care unit (iNICU). IEEE Access.

[33] Feichtenhofer, C., Fan, H., Malik, J. & He, K. Slowfast networks for video recognition. In *Proceedings of the IEEE/CVF International Conference on Computer Vision*, 6202–6211, (IEEE, 2019).

[34] Carreira, J. & Zisserman, A. Quo vadis, action recognition? a new model and the kinetics dataset. In *Proceedings of the IEEE Conference on Computer Vision and Pattern Recognition*, 6299–6308 (IEEE, 2017).

[35] Bai Q (2023). X3dfast model for classifying dairy cow behaviors based on a two-pathway architecture. Sci. Rep..

[36] Yang, D. et al. Aide: A vision-driven multi-view, multi-modal, multi-tasking dataset for assistive driving perception. In *Proceedings of the IEEE/CVF International Conference on Computer Vision*, 20459–20470, (IEEE, 2023).

[37] Zhang, C. L., Wu, J. & Li, Y. ActionFormer: Localizing moments of actions with transformers. In *European Conference on Computer Vision*, volume 13664 of *LNCS*, 492–510 (Springer, 2022).

[38] Vaswani, A. et al. Attention is all you need. In *Advances in Neural Information Processing Systems*, vol. 30 (Curran Associates, Inc., 2017).

[39] Hu, M. et al. Nurvid: A large expert-level video database for nursing procedure activity understanding. In *Thirty-seventh Conference on Neural Information Processing Systems Datasets and Benchmarks Track* (NeurIPS, 2023).

[40] Loshchilov, I. & Hutter, F. Decoupled weight decay regularization. In *International Conference on Learning Representations* (2019).

[41] Liu M, Nie L, Wang Y, Wang M, Rui Y (2023). A survey on video moment localization. ACM Comput. Surv..

[42] Vahdani E, Tian Y (2022). Deep learning-based action detection in untrimmed videos: A survey. IEEE Trans. Pattern Anal. Mach. Intell..

