# Supplementary material for: Deep learning to quantify care manipulation activities in neonatal intensive care units
Source: NPJ Digit Med. 2024 Jun 27;7:172. doi: 10.1038/s41746-024-01164-y (PMC11211355; doi:10.1038/s41746-024-01164-y)
Supplement: Supplementary file 1 — Supplementary Information [file 41746_2024_1164_MOESM1_ESM.pdf]

Supplementary Information

Supplementary Table 1. Results for care manipulation activity detection at tIOU=0.5. We report results for activities across splits with tIoU a threshold of 0.5 (corresponding to Figure 1 in the main paper). Precision and recall are reported based on the best F1 scores. Average precision is computed as the area under the precision-recall curve.

**Metrics@ tIOU=0.5**	**Action**	**Split Num**					**Average**
		**1**	**2**	**3**	**4**	**5**	
**Precision**	Diaper Change	0.738	0.727	0.689	0.711	0.773	0.728
	Feeding	0.857	0.875	0.878	0.921	0.780	0.862
	Patting	0.725	0.957	0.739	0.970	0.914	0.861
	Average	*0.773*	*0.853*	*0.769*	*0.867*	*0.822*	*0.817*
**Recall**	Diaper Change	0.689	0.727	0.646	0.667	0.756	0.697
	Feeding	0.714	0.622	0.935	0.814	0.762	0.769
	Patting	0.725	0.537	0.810	0.800	0.800	0.734
	Average	*0.709*	*0.629*	*0.797*	*0.760*	*0.773*	*0.734*
**F1 Score**	Diaper Change	0.713	0.727	0.667	0.688	0.764	0.712
	Feeding	0.779	0.727	0.906	0.864	0.771	0.809
	Patting	0.725	0.688	0.773	0.877	0.853	0.783
	Average	*0.739*	*0.714*	*0.782*	*0.810*	*0.796*	*0.768*
**Average Precision**	Diaper Change	0.670	0.750	0.620	0.660	0.760	0.692
	Feeding	0.810	0.680	0.890	0.860	0.760	0.800
	Patting	0.710	0.670	0.740	0.830	0.810	0.752
	Average	*0.730*	*0.700*	*0.750*	*0.783*	*0.777*	*0.748*

Supplementary Table 2. Results for care manipulation activity detection at tIOU=0.75. We report results for activities across 5 cross validation splits at a tIoU threshold of 0.75 (corresponding to Figure 4). Precision and recall are reported based on the best F1 scores. Average precision is computed as the area under the precision-recall curve.

**Metrics@ tIOU=0.75**	**Action**	**Split Num**					**Average**
		**1**	**2**	**3**	**4**	**5**	
**Precision**	Diaper Change	0.571	0.667	0.479	0.568	0.478	0.553
	Feeding	0.829	0.844	0.875	0.842	0.636	0.805
	Patting	0.650	0.957	0.743	0.970	0.800	0.824
	Average	*0.683*	*0.823*	*0.699*	*0.793*	*0.638*	*0.727*
**Recall**	Diaper Change	0.533	0.455	0.479	0.438	0.489	0.479
	Feeding	0.690	0.600	0.761	0.744	0.667	0.692
	Patting	0.650	0.537	0.619	0.800	0.700	0.661
	Average	*0.624*	*0.531*	*0.620*	*0.661*	*0.619*	*0.611*
**F1 Score**	Diaper Change	0.552	0.541	0.479	0.494	0.484	0.510
	Feeding	0.753	0.701	0.814	0.790	0.651	0.742
	Patting	0.650	0.688	0.675	0.877	0.747	0.727
	Average	*0.652*	*0.643*	*0.656*	*0.720*	*0.627*	*0.660*
**Average Precision**	Diaper Change	0.410	0.410	0.300	0.400	0.400	0.384
	Feeding	0.770	0.630	0.780	0.730	0.630	0.708
	Patting	0.590	0.620	0.580	0.800	0.630	0.644
	Average	*0.590*	*0.553*	*0.553*	*0.643*	*0.553*	*0.578*

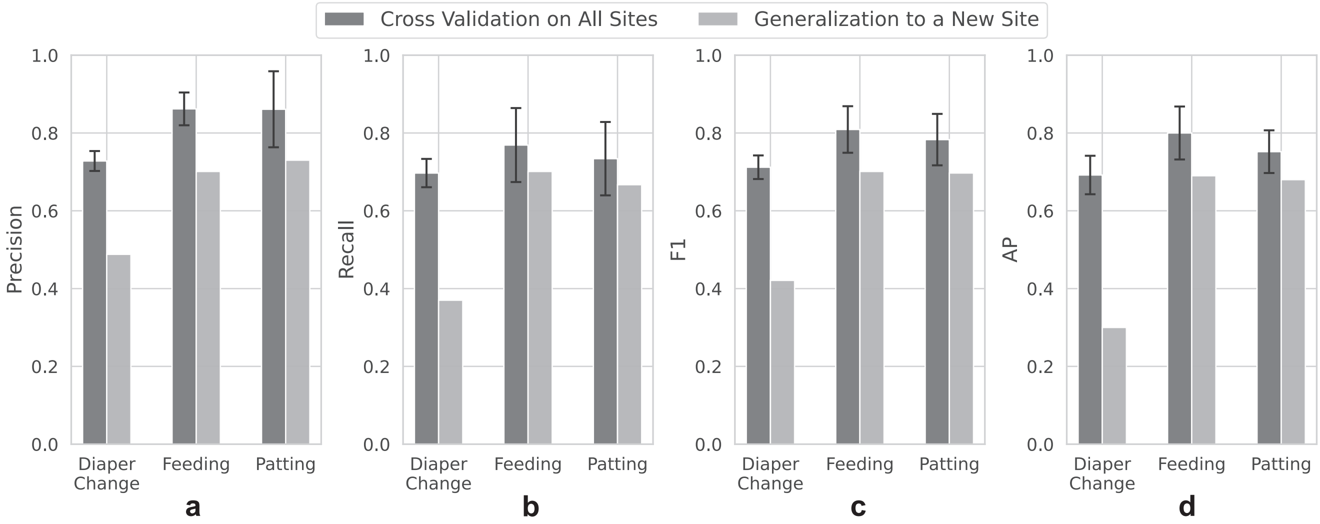

Supplementary Figure 1. Generalizability of our method for detecting care manipulation activities. In this experiment, our method was trained on videos from one site (an urban NICU with 223 videos), and subsequently evaluated on videos from a different site (a rural NICU with 107 videos). The results (in light gray) were further compared against those from cross-validation (5 splits) performed on aggregated data from both sites (in dark gray and with error bars). Minor performance declines were observed for feeding and patting activities, whereas a larger degradation was noted for diaper change. This variation in performance might be attributed to the difference in procedural execution and the pace at which these activities were conducted in NICUs situated in urban versus rural settings.

Supplementary Table 3. Variation in heart rate and SpO_2 levels when comparing (a) before (baseline) and during an activity; and (b) before (baseline) and post an activity. P-values using paired t-tests are also reported in brackets.

Manipulation Activity	Method	Heart Rate (beats per minute)		SpO_2 (%)	
		During *vs.* Before	Post *vs.* Before	During *vs.* Before	Post *vs.* Before
Diaper Change	alg	+6.16 ($p<0.01$, n=114)	-1.04 (p=0.28, n=117)	-0.31 (p=0.07, n=113)	+0.66 (p=0.09, n=117)
	human	+5.60 ($p<0.01$, n=181)	-0.75 (p=0.36, n=190)	-0.42 (p=0.07, n=181)	+0.36 (p=0.09, n=190)
Tube Feeding	alg	-0.91 (p=0.46, n=106)	+0.07 (p=0.95, n=110)	-0.04 (p=0.93, n=106)	-0.07 (p=0.87, n=110)
	human	+0.36 (p=0.75, n=129)	+1.58 (p=0.11, n=139)	+0.15 (p=0.65, n=129)	-0.17 (p=0.63, n=139)
Patting	alg	+1.62 (p=0.29, n=70)	+1.15 (p=0.18, n=130)	-0.24 (p=0.56, n=70)	+0.08 (p=0.78, n=130)
	human	+2.04 (p=0.16, n=78)	+1.37 (p=0.07, n=148)	-0.67 (p=0.13, n=78)	-0.28 (p=0.33, n=148)

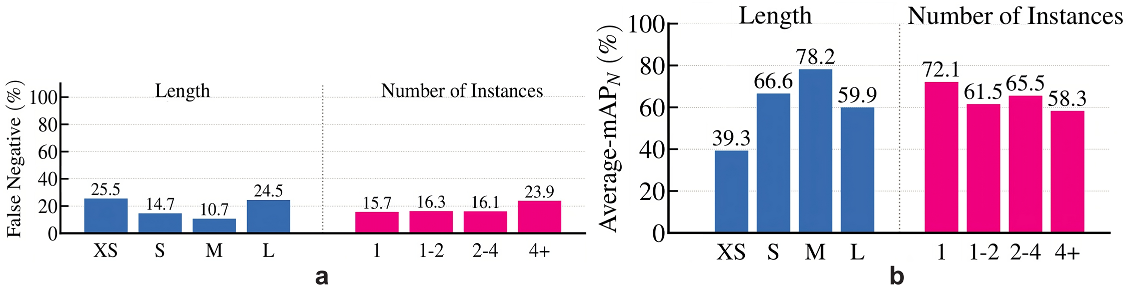

Supplementary Figure 2. Diagnostic analyses of activity detection results using the tool from Alwassel et al.[1]. False negative rates (**a**) and mean average precision (**b**) are broken down based on the duration of the activities (length) and the number of instances per video. XS, S, M, and L respectively represent activities with duration 0s-30s, 30s-180s, 180s-360s, and >360s.

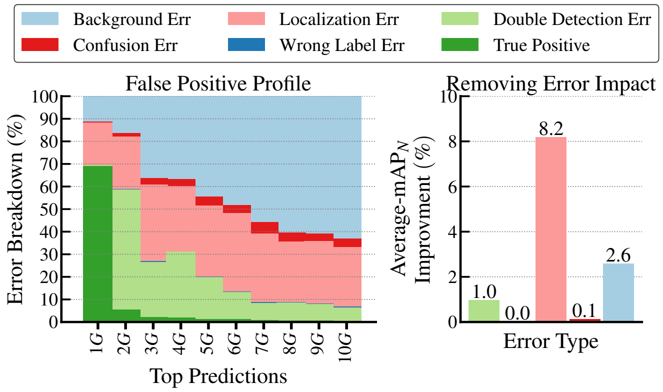

Supplementary Figure 3. Error analyses of activity detection results using the tool from Alwassel et al.[1]. False positive errors are categorized into (1) background error, where a prediction does not majorly overlap (less than the tIoU threshold) with any of the ground-truth activities; (2) localization error, where a prediction with correct label overlaps with a ground-truth yet fails to pass the tIoU threshold; (3) double detection error, where two predictions with correct label satisfying the tIoU threshold are matched to the same ground-truth; (4) confusion error, a prediction with incorrect label overlaps with a ground-truth yet fails to pass the tIoU threshold; and (5) wrong label error, where a prediction with incorrect label majorly overlaps with a ground-truth. **Left**: The false positive profile of our method. The profile shows a breakdown of false positive errors and true positives with varying number of ground-truth activities (e.g., 1G indicates one ground-truth activity per video). **Right**: The impact of different false positive error types on the mean average precision.

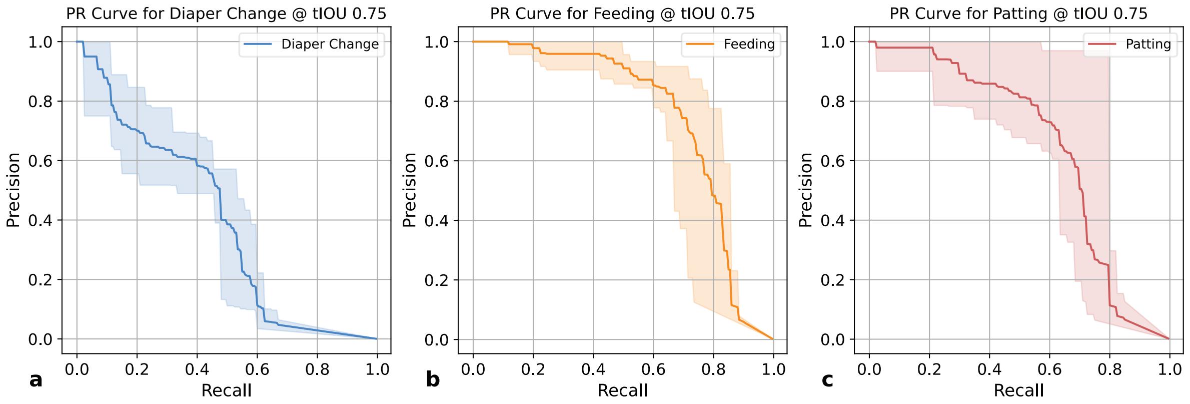

Supplementary Figure 4. Activity detection results. Precision-recall (PR) curves for three manipulation activities (diaper change (**a**), feeding (**b**), and patting (**c**)) at a tIOU threshold of 0.75. Lighter bands indicate the variations across five test splits.

Supplementary Table 4. A comparative analysis of the results in detecting care manipulation activities. ActionFormer, employed in our method, was compared to most recent methods designed for detecting activities in video (row 1-3), using the same video representation (SlowFast[2]). Further, the effects of fine-tuning was studied (row 3-4). Results were averaged across 5 splits and reported at tIOU = 0.5 and 0.75. ActionFormer outperformed other recent methods, attesting our design. Notably, fine-tuning yielded significant improvements under the more stringent criteria with tIoU=0.75.

Method	Finetuned Features	Metrics @tIOU=0.5				Metrics @tIOU=0.75			
		Precision	Recall	F1-Score	AP	Precision	Recall	F1-Score	AP
TadTR[3]	✓	0.606	0.496	0.542	0.447	0.410	0.299	0.335	0.222
TriDet[4]	✓	0.816	0.728	0.764	0.771	0.705	0.565	0.622	0.559
ActionFormer[5]	✓	0.817	0.734	0.768	0.748	0.727	0.611	0.660	0.578
ActionFormer[5]	×	0.835	0.735	0.778	0.764	0.704	0.582	0.633	0.555

Supplementary Table 5. The architecture of ActionFormer for localizing manipulation activities in videos. The model consists of (1) a Transformer encoder (first row block) and (2) a lightweight convolutional decoder with the classification / regression heads (last row block). For each layer, the layer name, layer parameters, the input to the layer, the output feature size, and output regression ranges (if applicable) are listed. For convolutional layers, k is the kernel size of 1D convolutions and s is the stride, and c_i, c_o is the input and output feature channel, respectively. For the Transformer units, ds is the downsampling ratio. T is the temporal length of input sequence and D is the input feature dimension. For classification head, the output dimension is the number of manipulation activity categories. For regression head, the output dimension is 2, i.e., distances to an activity's onset and offset.

	Name	Layer	Input	Output Size (T × D)	Regression Range
encoder	input clip	-	-	T×D	-
	projection1	conv k=3, s=1 (c_i = D, c_o = 512)	input clip	T × 512	-
	projection2	conv k=3, s=1 (c_i = 512, c_o = 512)	projection1	T × 512	-
	transformer0	Transformer Unit, ds=1	projection2	T × 512	-
	transformer1	Transformer Unit, ds=1	transformer0	T × 512	[0, 4)
	transformer2	Transformer Unit, ds=2	transformer1	T/2 ×512	[2, 8)
	transformer3	Transformer Unit, ds=2	transformer2	T/4 ×512	[4, 16)
	transformer4	Transformer Unit, ds=2	transformer3	T/8 ×512	[8, 32)
	transformer5	Transformer Unit, ds=2	transformer4	T/16 ×512	[16, 64)
	transformer6	Transformer Unit, ds=2	transformer5	T/32 ×512	[32, 128)
	transformer7	Transformer Unit, ds=2	transformer6	T/64 ×512	[64, 256)
	transformer8	Transformer Unit, ds=2	transformer7	T/128 ×512	[128, +∞)
decoder (heads)	cls / reg nets	conv k=3, s=1 (c_i = 512, c_o = 512)	transformer1,...,transformer8	[T/128×512,..., T×512]	-
		conv k=3, s=1 (c_i = 512, c_o = 512)	transformer1,...,transformer8	[T/128×512,..., T×512]	-
		conv k=3, s=1 (c_i = 512, c_o = output)	transformer1,...,transformer8	[T/128×output,..., T×output]	-

Supplementary Table 6. Ablation study of the model architecture. Parameters determining the model architecture, including the number of transformer blocks (# Layers), the embedding dimension (Emb Dim), and the scaling factor of the regression range (Reg Range Scaling), were studied. Results were averaged across 5 splits and reported at tIOU = 0.5 and 0.75. Rows highlighted in **bold** corresponds to architecture used in our main experiments.

# Layers	Emb Dim	Reg Range Scaling	Metrics @tIOU=0.5				Metrics @tIOU=0.75			
			Precision	Recall	F1-score	AP	Precision	Recall	F1-score	AP
1	512	1×	0.570	0.570	0.560	0.498	0.361	0.331	0.323	0.225
2	512	1×	0.670	0.663	0.659	0.592	0.476	0.440	0.450	0.344
3	512	1×	0.758	0.715	0.728	0.698	0.584	0.521	0.543	0.444
4	512	1×	0.774	0.733	0.751	0.722	0.623	0.547	0.579	0.484
5	512	1×	0.818	0.726	0.765	0.761	0.669	0.580	0.617	0.533
6	512	1×	0.811	0.751	0.776	0.759	0.713	0.610	0.651	0.569
7	**512**	**1×**	**0.817**	**0.734**	**0.768**	**0.748**	**0.727**	**0.611**	**0.660**	**0.578**
8	512	1×	0.831	0.734	0.777	0.774	0.715	0.616	0.659	0.595
7	*128*	1×	0.826	0.745	0.779	0.772	0.704	0.603	0.645	0.561
7	*256*	1×	0.838	0.737	0.780	0.769	0.703	0.611	0.650	0.569
**7**	***512***	**1×**	**0.817**	**0.734**	**0.768**	**0.748**	**0.727**	**0.611**	**0.660**	**0.578**
7	*1024*	1×	0.771	0.714	0.738	0.735	0.674	0.560	0.607	0.539
7	512	*0.5×*	0.821	0.728	0.769	0.768	0.685	0.549	0.606	0.523
**7**	**512**	***1×***	**0.817**	**0.734**	**0.768**	**0.748**	**0.727**	**0.611**	**0.660**	**0.578**
7	512	*2×*	0.819	0.718	0.761	0.739	0.680	0.583	0.623	0.537

Supplementary Table 7. Detailed statistics for the cross validation splits. Sample size, average duration, and average frequency of activities across 5 cross validation data splits.

Split		Diaper Change			Tube Feeding			Patting		
		Sample Size	Avg Duration (seconds)	Avg Freq (# per hour)	Sample Size	Avg Duration (seconds)	Avg Freq (# per hour)	Sample Size	Avg Duration (seconds)	Avg Freq (# per hour)
S1	train	185	224.00	1.10	175	111.52	1.14	163	32.52	1.15
	test	45	244.82	1.12	42	119.31	1.13	40	33.93	1.26
S2	train	186	229.33	1.11	173	112.75	1.14	162	31.51	1.18
	test	44	222.77	1.09	44	114.11	1.12	41	37.85	1.13
S3	train	182	233.65	1.11	171	114.32	1.13	161	33.83	1.16
	test	48	206.94	1.08	46	108.24	1.17	42	28.81	1.18
S4	train	182	231.30	1.10	174	111.67	1.14	163	32.94	1.19
	test	48	215.85	1.10	43	118.49	1.11	40	32.18	1.08
S5	train	185	222.23	1.10	175	114.89	1.13	163	33.17	1.19
	test	45	252.09	1.13	42	105.26	1.15	40	31.28	1.19

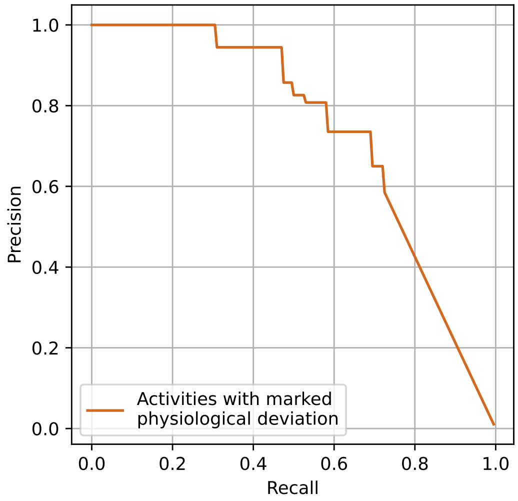

Supplementary Figure 5. Detecting activities with marked deviations in physiological response. Precision-Recall (PR) curve for detecting events with marked deviations in physiological response at a tIOU threshold of 0.5. A total of 36 such events were derived from our data. The results were aggregated across all test splits.

Supplementary References

1. Alwassel, H., Heilbron, F. C., Escorcia, V. & Ghanem, B. Diagnosing error in temporal action detectors. In *Proceedings of the European conference on computer vision (ECCV)*, 256–272 (”Springer International Publishing”, 2018).
2. Feichtenhofer, C., Fan, H., Malik, J. & He, K. Slowfast networks for video recognition. In *Proceedings of the IEEE/CVF International Conference on Computer Vision*, 6202–6211 (2019).
3. Liu, X. *et al.* End-to-end temporal action detection with transformer. *IEEE Transactions on Image Process.* **31**, 5427–5441 (2022).
4. Shi, D. *et al.* TriDet: Temporal Action Detection with Relative Boundary Modeling. In *2023 IEEE/CVF Conference on Computer Vision and Pattern Recognition (CVPR)*, 18857–18866 (IEEE, 2023).
5. Zhang, C.-L., Wu, J. & Li, Y. ActionFormer: Localizing moments of actions with transformers. In *European Conference on Computer Vision*, vol. 13664 of *LNCS*, 492–510 (Springer, 2022).
