# Supplementary material for: Deep learning to quantify care manipulation activities in neonatal intensive care units
Source: NPJ Digit Med. 2024 Jun 27;7:172. doi: 10.1038/s41746-024-01164-y (PMC11211355; doi:10.1038/s41746-024-01164-y)
Supplement: Supplementary file 2 — Reporting Summary [file 41746_2024_1164_MOESM2_ESM.pdf]

nature portfolio

Corresponding author(s): Yin Li

Last updated by author(s): Apr 25, 2024

Reporting Summary

Nature Portfolio wishes to improve the reproducibility of the work that we publish. This form provides structure for consistency and transparency in reporting. For further information on Nature Portfolio policies, see our Editorial Policies and the Editorial Policy Checklist.

Statistics

For all statistical analyses, confirm that the following items are present in the figure legend, table legend, main text, or Methods section.

n/a	Confirmed	
☐	☒	The exact sample size (n) for each experimental group/condition, given as a discrete number and unit of measurement
☐	☒	A statement on whether measurements were taken from distinct samples or whether the same sample was measured repeatedly
☐	☒	The statistical test(s) used AND whether they are one- or two-sided<br>*Only common tests should be described solely by name; describe more complex techniques in the Methods section.*
☒	☐	A description of all covariates tested
☒	☐	A description of any assumptions or corrections, such as tests of normality and adjustment for multiple comparisons
☐	☒	A full description of the statistical parameters including central tendency (e.g. means) or other basic estimates (e.g. regression coefficient) AND variation (e.g. standard deviation) or associated estimates of uncertainty (e.g. confidence intervals)
☐	☒	For null hypothesis testing, the test statistic (e.g. F, t, r) with confidence intervals, effect sizes, degrees of freedom and P value noted<br>*Give P values as exact values whenever suitable.*
☒	☐	For Bayesian analysis, information on the choice of priors and Markov chain Monte Carlo settings
☒	☐	For hierarchical and complex designs, identification of the appropriate level for tests and full reporting of outcomes
☒	☐	Estimates of effect sizes (e.g. Cohen's d, Pearson's r), indicating how they were calculated

Our web collection on statistics for biologists contains articles on many of the points above.

Software and code

Policy information about availability of computer code

Data collection	Data was collected using a proprietary software (iNICU) from Child Health Imprints Inc. The design of this software was described in the paper (Singh, H., Kaur, R., Gangadharan, A., Pandey, A. K., Manur, A., Sun, Y., ... & Kumar, P. (2018). Neo-bedside monitoring device for integrated neonatal intensive care unit (iNICU). IEEE Access, 7, 7803-7813).
Data analysis	Our analysis used a collection of open-source software, including PyTorch 1.12.1, Pandas 1.4.3, NumPy 1.23.4, ActionFormer, and SlowFast 1.0.

For manuscripts utilizing custom algorithms or software that are central to the research but not yet described in published literature, software must be made available to editors and reviewers. We strongly encourage code deposition in a community repository (e.g. GitHub). See the Nature Portfolio guidelines for submitting code & software for further information.

Data

Policy information about availability of data

All manuscripts must include a data availability statement. This statement should provide the following information, where applicable:

- Accession codes, unique identifiers, or web links for publicly available datasets
- A description of any restrictions on data availability
- For clinical datasets or third party data, please ensure that the statement adheres to our policy

The datasets considered in this study are not publicly available due to the sensitive and identifiable nature of the data, parental consent and restrictions of the ethics protocol to protect the privacy of preterm infants involved in the study.

Research involving human participants, their data, or biological material

Policy information about studies with human participants or human data. See also policy information about sex, gender (identity/presentation), and sexual orientation and race, ethnicity and racism.

Reporting on sex and gender	N/A
Reporting on race, ethnicity, or other socially relevant groupings	N/A
Population characteristics	Preterm infants, average age: 33 weeks
Recruitment	Data were collected from 27 neonates admitted to two NICUs in India during 2020.
Ethics oversight	Institutional review board at the following sites has approved the study: (1) Apollo Cradle in Moti Nagar, Delhi, India; and (2) Kalawati Hospital, Rewari, India

Note that full information on the approval of the study protocol must also be provided in the manuscript.

Field-specific reporting

Please select the one below that is the best fit for your research. If you are not sure, read the appropriate sections before making your selection.

☒ Life sciences ☐ Behavioural & social sciences ☐ Ecological, evolutionary & environmental sciences

For a reference copy of the document with all sections, see nature.com/documents/nr-reporting-summary-flat.pdf

Life sciences study design

All studies must disclose on these points even when the disclosure is negative.

Sample size	The sample size was determined by the resources available for collecting and annotating videos.
Data exclusions	6 videos with incorrect/anomalous annotations were excluded from the analysis.
Replication	Results were successfully reproduced by running the same analysis on the dataset with multiple computers.
Randomization	Random stratified 5 fold cross validation was performed using SciKit learn package of Python.
Blinding	Blinding is not relevant to our study due to the randomization.

Reporting for specific materials, systems and methods

We require information from authors about some types of materials, experimental systems and methods used in many studies. Here, indicate whether each material, system or method listed is relevant to your study. If you are not sure if a list item applies to your research, read the appropriate section before selecting a response.

Materials & experimental systems

n/a	Involved in the study
☒	☐ Antibodies
☒	☐ Eukaryotic cell lines
☒	☐ Palaeontology and archaeology
☒	☐ Animals and other organisms
☐	☒ Clinical data
☒	☐ Dual use research of concern
☒	☐ Plants

Methods

n/a	Involved in the study
☒	☐ ChIP-seq
☒	☐ Flow cytometry
☒	☐ MRI-based neuroimaging

Clinical data

Policy information about clinical studies

All manuscripts should comply with the ICMJE guidelines for publication of clinical research and a completed CONSORT checklist must be included with all submissions.

Clinical trial registration

Provide the trial registration number from ClinicalTrials.gov or an equivalent agency.

Study protocol

Note where the full trial protocol can be accessed OR if not available, explain why.

Data collection

Describe the settings and locales of data collection, noting the time periods of recruitment and data collection.

Outcomes

Describe how you pre-defined primary and secondary outcome measures and how you assessed these measures.

Plants

Seed stocks

Report on the source of all seed stocks or other plant material used. If applicable, state the seed stock centre and catalogue number. If plant specimens were collected from the field, describe the collection location, date and sampling procedures.

Novel plant genotypes

Describe the methods by which all novel plant genotypes were produced. This includes those generated by transgenic approaches, gene editing, chemical/radiation-based mutagenesis and hybridization. For transgenic lines, describe the transformation method, the number of independent lines analyzed and the generation upon which experiments were performed. For gene-edited lines, describe the editor used, the endogenous sequence targeted for editing, the targeting guide RNA sequence (if applicable) and how the editor was applied.

Authentication

Describe any authentication procedures for each seed stock used or novel genotype generated. Describe any experiments used to assess the effect of a mutation and, where applicable, how potential secondary effects (e.g. second site T-DNA insertions, mosiacism, off-target gene editing) were examined.
